# Acetylcholinesterase as a Biomarker in Environmental and Occupational Medicine: New Insights and Future Perspectives

**DOI:** 10.1155/2013/321213

**Published:** 2013-07-11

**Authors:** Maria Giulia Lionetto, Roberto Caricato, Antonio Calisi, Maria Elena Giordano, Trifone Schettino

**Affiliations:** Department of Biological and Environmental Sciences and Technologies (DiSTeBA), University of Salento, Via Prov.le Lecce-Monteroni, 73100 Lecce, Italy

## Abstract

Acetylcholinesterase (AChE) is a key enzyme in the nervous system. It terminates nerve impulses by catalysing the hydrolysis of neurotransmitter acetylcholine. As a specific molecular target of organophosphate and carbamate pesticides, acetylcholinesterase activity and its inhibition has been early recognized to be a human biological marker of pesticide poisoning. Measurement of AChE inhibition has been increasingly used in the last two decades as a biomarker of effect on nervous system following exposure to organophosphate and carbamate pesticides in occupational and environmental medicine. The success of this biomarker arises from the fact that it meets a number of characteristics necessary for the successful application of a biological response as biomarker in human biomonitoring: the response is easy to measure, it shows a dose-dependent behavior to pollutant exposure, it is sensitive, and it exhibits a link to health adverse effects. The aim of this work is to review and discuss the recent findings about acetylcholinesterase, including its sensitivity to other pollutants and the expression of different splice variants. These insights open new perspective for the future use of this biomarker in environmental and occupational human health monitoring.

## 1. Introduction

Biological markers (biomarkers) were early defined as “cellular, biochemical or molecular alterations that are measurable in biological media such as human tissues, cells, or fluids” [1]. More recently, the definition includes biological characteristics that can be objectively measured and evaluated as indicator of normal biological processes, pathogenic processes, or pharmacological responses to a therapeutic intervention [2].

**Figure 1:**
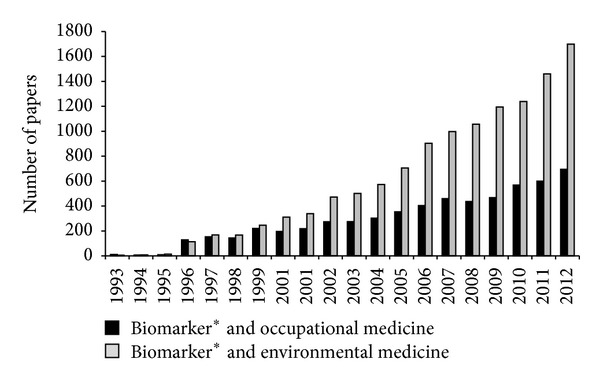
Number of papers published in the last 20 years. The research was carried out on Scopus by using two research queries, respectively: (1) “biomarker*” and “occupational medicine,” (2) “biomarker*” and “Environmental medicine” (Scopus, April 2013).

Biomarkers are useful tools in a variety of fields, including medicine, environmental health, toxicology, developmental biology, and basic scientific research. In the last two decades a growing interest towards biomarkers has been recorded in occupational and environmental medicine, as observed in Figure 1, where the trend of the number of papers published in these fields in the last 20 years is reported. The interest in biomarkers in occupational and environmental medicine parallels the development of human biomonitoring which is defined as the repeated, controlled measurement of chemical or biomarkers in fluids, tissues, or other accessible samples from subjects currently exposed or had been exposed in the past or to be exposed to chemical, physical, or biological risk factors in the workplace and/or the general environment [3]. Human biomonitoring is a valuable tool in exposure estimation of selected populations and currently used in surveillance programs all over the world.

Biomarkers used in environmental and occupational human health monitoring can be distinguished into three classes: biomarker of exposure, effect, and susceptibility [4]. Biomarkers of exposure involve measurement of parent compound, metabolites and reflect the dose of exposure. Biomarkers of effect are a measurable biochemical, physiological, and behavioral alteration within an organism that can be recognized as associated with an established or possible health impairment or disease. Biomarkers of susceptibility indicate an inherent or acquired ability of an organism to respond to specific exposure [3].

In the last two decades a variety of biomarkers have been used to study worker populations, and these studies have contributed at different levels to the improvement of occupational health. On the basis of this success there is a continued need to develop and apply biomarkers as useful tools for providing real-time detection of exposure to hazardous substances in the workplace and in the general environment [4]. 

One of the early biomarkers characterized in human environmental exposure is represented by the inhibition of the enzyme acetylcholinesterase (AChE) as biomarker of effect on nervous system following complex exposure to organophosphorus compounds.

The present work aims to review and discuss the recent findings on this biomarker in relation to the current and future use in environmental and occupational human health monitoring. 

## 2. AChE: General Features

AChE belongs to the family of cholinesterases (ChEs), which are specialized carboxylic ester hydrolases that break down esters of choline. Cholinesterase class includes AChE which hydrolyzes the neurotransmitter acetylcholine and pseudocholinesterase or butyrylcholinesterase (BChE) which utilizes butyrylcholine as substrate. AChE is mainly found at neuromuscular junctions and cholinergic synapses in the central nervous system. Here, AChE hydrolyzes acetylcholine into choline and acetate after activation of acetylcholine receptors at the postsynaptic membrane. AChE activity serves to terminate synaptic transmission, preventing continuous nerve firings at nerve endings. Therefore, it is essential for the normal functioning of the central and peripheral nervous system. AChE is also found on the red blood cell membranes, where it constitutes the Yt blood group antigen [5] also known as Cartwright. It helps to determine a person's blood type, but the physiological function on erythrocyte membrane is to date unknown [5]. BChE is found in plasma and its physiological function in blood remains still unknown [6].

The AChE molecule is composed of two different protein domains: a large catalytic domain of about 500 residues and a small C-terminal peptide of less than 50 residues. AChE is a product of a single gene [7] which is expressed in different tissues in different splicing forms. Alternative splicing in the 3′ terminus of AChE pre-mRNA produces three variants: the primary, “synaptic” AChE-S (otherwise known as “tailed,” AChE-T) [8], the stress-induced, soluble (readthrough) AChE-R variant, and the erythrocytic AChE-E [9]. These isoforms share a similar catalytic domain but differ in their C-terminal domain, which influences their molecular form and localization and confers specific features [10]. “Synaptic” AChE-S constitutes the principal multimeric enzyme in brain and muscle. It is typically tetrameric and membrane bound in the synapse. The soluble, monomeric “readthrough” AChE-R is induced under chemical and physical stress; the erythrocytic AChE-E is a glycophosphatidylinositol- (GPI-) linked dimer targeted to the plasma membrane of erythrocyte and lymphocytes [11]. AChE-S and AChE-R have been described also in peripheral blood cells [12].

The active site of AChE includes two subsites: the anionic site and the esteratic subsite. The anionic subsite is the binding site for the positive quaternary amine of acetylcholine. The esteratic subsite is the site where acetylcholine is hydrolyzed to acetate and choline. The hydrolysis of the carboxyl ester leads to the formation of an acyl-enzyme and free choline. Then, the acyl-enzyme undergoes nucleophilic attack by a water molecule, liberating acetic acid and regenerating the free enzyme [13].

## 3. Organophosphorus and Carbamate Compounds as Specific Inhibitors of AChE

Organophosphorus and carbamate pesticides are known to be specific inhibitors of aceylcholinesterase catalytic activity [14]. They have become the most widely used pesticides today since the removal of organochlorine pesticides from use. Organophosphorus and carbamate compounds bind with variable affinity to the esteratic site by phosphorylation or decarbamylation, respectively, and inactivate the enzyme. Organophosphorus compounds are considered to be functionally irreversible inhibitors of AChE, since the time necessary to liberate the enzyme from inhibition may be in excess of the time required for synthesis of new AChE. Carbamates, on the other hand, have a fairly rapid decarbamylation step so that substantial recovery of the enzyme can occur in a finite period of time. The hydrolysis rate of the intermediate phosphorylated or carbamated enzyme is not the only factor contributing to the toxicity of these pesticides. The affinity of the serine-hydroxyl group in the active site (esteratic site) for the inhibitor is another important aspect to be considered. Some compounds have a direct effect on the enzyme, while others such as parathion or chlorpyrifos, that have little capacity to directly inhibit AChE, are metabolically activated by cytochromes P450 to form potent AChE inhibitors referred to as “oxygen analogs” or “oxons.” While it has been known that these oxons inhibit AChE through phosphorylation of Ser-203, the details of the interactions between these oxons and the enzyme are unclear. Recent results [15]suggest that the interactions of chlorpyrifos oxon with AChE are complex and may involve the binding of this oxon to a secondary site on the enzyme.

Organophosphate and carbamate pesticides are widely used for pest control on crops in agriculture and on livestock and for residential uses, including insect control in domestic and garden uses. Organophosphorus pesticide residues have been detected at permissible (and sometimes impermissible) levels in many agricultural products; therefore low-level dietary exposures to organophosphorus pesticides are likely. Occupational exposure occurs at all stages of pesticide formulation, manufacture, and production and implies exposure to complex mixtures of different types of these compounds. In general, occupational exposures to organophosphorus pesticides dwarf environmental exposures [16]; however, special populations, such as farm-worker children, may receive higher exposures. Large amounts of these compounds are released into the environment and many of them exert their effect also on nontarget organisms [17–20], being a potential hazard for human health and the environment. The residues of organophosphate and carbamate from agricultural practices are able to infiltrate through the soil into surface water because of their water solubility [21]. As a consequence of their wide diffusion there, residues have been detected in food [22], ground and drinking water [23], natural surface waters [24], and marine organisms [25]. Therefore, all people are inevitably exposed to these compounds and/or their degradation products through environmental contamination or occupational use in air, water, and food. These pollutants cannot be easily detected by chemical analysis because of their relative short life in the environment; on the other hand their products of environmental degradation can be very harmful, retaining anticholinesterase activity [26]. 

As recently outlined by Black and Read [27] the AChE inhibition by organophosphorus compounds arouses a certain interest also in relationship to the problem of exposure to chemical warfare agents, such as organophosphorus nerve agents [28]. These are the most toxic chemical warfare agents that are known to have been produced, stockpiled, and weaponized. Their development, production, stockpiling, and use are prohibited under the terms of the Chemical Weapons Convention, and, together with their precursors, are subject to strict controls and verification procedures [27]. The first confirmed use of organophosphorus nerve agents in warfare was by Iraq in the conflict with Iran (United Nations, 1984) and by Iraq against Kurdish population. More recently there has been a perceived increased risk of some terrorist groups using nerve agents [27].

## 4. AChE Inhibition as a Biomarker of Effect in Occupational and Environmental Medicine

**Figure 2:**
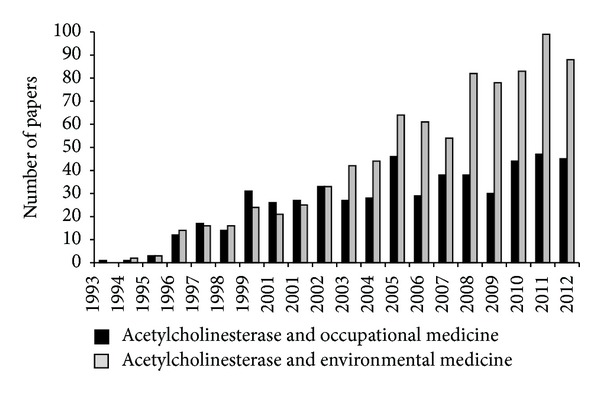
Number of papers published in the last 20 years. The research was carried out on Scopus by using two research queries, respectively: (1) “AChE” and “occupational medicine,” (2) “AChE” and “Environmental medicine” (Scopus, April 2013).

As a molecular target of organophosphorus and carbamate compounds, AChE measurement in the blood was early recognized to be a human biological marker of effect for these molecules and emerged as a diagnostic tool in the biomedical area. As observed in Figure 2 in the last two decades a growing interest in AChE as a biomarker in occupational and environmental medicine has been observed, as indicated by the growing number of papers in these fields.

Today measurement of ChE levels in blood is the conventional method of assessing the degree of occupational exposure to organophosphate pesticides in exposed environments (e.g., environments concerned with pesticide production and use) during the periodic statutory medical surveillance in several countries [29]. Intervention levels have been established, for example, in Sweden: if AChE inhibition (calculated with respect to the individual preexposure level–baseline) is 25%, a second measurement has to be carried out. If the decrease in ACHE activity is confirmed, exposure has to be avoided for 14 days [4].

Blood cholinesterase measurement is also useful as a primary biomarker in emergency medicine in cases of poisoning and accidental organophosphate or carbamate exposure [29–33]. In occupational and environmental medicine erythrocyte AChE and plasma or serum BChE are the two principal types of ChE measured in blood. Potential inhibition of AChE and BChE varies widely among the different organophosphorus compounds. Some organophosphate pesticides inhibit BChE more strongly than AChE. The inhibition of BChE is highly correlated with intensity and duration of higher exposure to a large group of organophosphate and carbamate pesticides [34]. However, BChE inhibition does not reflect the biological effects of organophosphate in the nervous system [35]. On the other hand AChE inhibition is more sensitive than BChE in the case of chronic exposure to organophosphate. In fact, AChE inhibition by organophosphate shows a lower recovery rate compared to BChE and this produces cumulative inhibitory effect on the AChE activity [36]. Unlike BChE, erythrocyte AChE inhibition mirrors the biological effects of organophosphate in the nervous system. Therefore, red blood cell measures of AChE are generally preferred over plasma measures of ChE activity because data on red blood cells may provide a better representation of the inhibition of the neural AChE. 

The success of the use of AChE inhibition as a biomarker of effect to organophosphate exposure arises from the fact that it meets a number of characteristics necessary for the successful application of a biological response as a biomarker in biomonitoring: the response is easy to measure, shows a dose-dependent behavior to pollutant exposure, is sensitive, and exhibits a link to health adverse effects.

The most widely used method for AChE activity measurement in blood is the Ellman method [37] based on the photometric determination of the chromogenic product coming from the reaction between acetylthiocholine (the substrate of the enzyme) and 5, 5-dithiobis-2-nitrobenzoic acid (DTNB, Ellman's reagent). This method is easy to use, employs relatively inexpensive equipment and the results are accurate and quantitative. Recently, the measurement of AChE inhibition in human saliva as a biomarker of effect for organophosphorus pesticide has been explored [37, 38]. In the last years the use of saliva as a diagnostic fluid for biomarker development has rapidly grown. The use of saliva for biomarker detection offers many advantages: saliva collection is noninvasive compared with phlebotomy, it is more acceptable to patients, and it does not carry the risk of needle-stick injuries [39]. These characteristics make the use of saliva suitable for medical surveillance and biological monitoring. AChE in human saliva is derived from salivary glandular cells, while BChE may be derived from microorganisms in the oral cavity [40]. Sayer et al. [41] demonstrated that AChE catalytic activity in saliva is stable at room temperature for up to 6 h. In a group of exposed pesticide factory workers, cholinesterase activity in saliva was found to be lower than the activity in healthy controls [42]. Henn et al. [43] suggested that saliva may be a useful indicator of potential neurotoxic effects from exposure to organophosphorus and carbamate pesticides but pointed out the need to further explore the factors affecting the high variability in the measures compared to blood AChE measurement. A study of Ng et al. [44] questioned the use of AChE in saliva as a biomarker for organophosphate compounds because of the low levels of AChE in saliva relative to erythrocytes and the weak correlation between the two measurements. Therefore, the use of AChE measurement as a biomarker of effect instead of blood measurement remains still debated.

**Figure 3:**
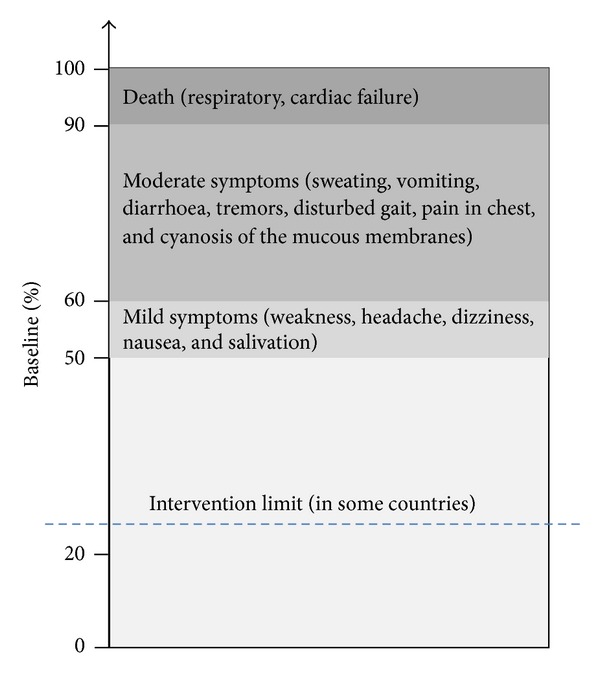
Relationship between AChE inhibition and health negative effects. Drawn on the basis of Maroni et al. [49] findings.

The use of a biomarker in biomonitoring requires the knowledge of the relationships between chemical exposure, biomarker responses and adverse effects. These aspects have been well established in the case of AChE. Several studies reported significant relationship between exposure to organophosphorus compounds and AChE inhibition in exposed worker populations [30, 45–48]. As regards the relationship between AChE inhibition and health negative effects, it is known that an inhibition of AChE between 50% and 60% elicits a dose-response pattern of relatively mild symptoms such as weakness, headache, dizziness, nausea, and salivation with a convalescence of 1–3 days (Figure 3). An inhibition of AChE between 60 and 90% produces moderate symptoms such as sweating, vomiting, diarrhoea, tremors, disturbed gait, pain in chest, and cyanosis of the mucous membranes which reverse within few weeks. At 90–100% inhibition, death from respiratory or cardiac failure occurs [49].

## 5. Sensitivity of AChE to Other Pollutants

In the last years, the inhibition of AChE from several chemical species other than organophosphate and carbamate pesticides including heavy metals, other pesticides, polycyclic aromatic hydrocarbons, detergents, and components of complex mixtures of contaminants has been increasingly reported in humans and other animals [50–54]. 

The potential of some metallic ions, such as Hg^2+^, Cd^2+^, Cu^2+^, and Pb^2+^, to depress the activity of AChE *in vitro* and/or *in vivo* conditions has been demonstrated in several studies on humans and animals [55–57]. Ademuyiwa et al. [57] studied the potential effect of lead on erythrocyte AChE activity during occupational exposure to this metal and suggested that erythrocyte AChE activity could be used as a biomarker of lead-induced neurotoxicity in occupational exposed subjects. 

AChE activity may also be affected by other pesticides from different chemical families, such as pyrethroids [58], triazines [59], and Paraquat [60]. Hernández et al. [61] suggested the usefulness of AChE as a biomarker of exposure in the surveillance of workers long-term exposed to pesticides other than organophosphate and carbamate.

Several findings also indicate the anticholinesterase effect of polycyclic aromatic hydrocarbons which are common environmental contaminants in surface waters, sediments, soils, and urban air. These compounds are formed during the incomplete combustion of fossil fuels, wood, and municipal waste incineration, from internal combustion engines. Kang and Fang [62] demonstrated that several polycyclic aromatic hydrocarbons inhibit AChE directly *in vitro*. The magnitude of the inhibition differs among the compounds tested and may be related to the number of aromatic rings in the molecule [63]. Interestingly, polycyclic aromatic hydrocarbons are able to inhibit AChE activity in an additive manner together with organophosphate, being noncompetitive inhibitors of AChE [63].

Recently, due to the growing interest in nanomaterials in various applications (e.g., electronics, biomedicine, catalysis, and material science) [64, 65], Wang et al. [66] explored the potential effects of nanoparticles on AChE activity *in vitro*. Different classes of nanoparticles, including metals, oxides, and carbon nanotubes (SiO_2_, TiO_2_, Al_2_O_3_, Al, Cu, carbon-coated copper, multiwalled carbon nanotubes, and single-walled carbon nanotubes), showed high affinity for AChE. Cu, Cu–C, multiwalled carbon nanotubes, and single-walled carbon nanotubes MWCNT, SWCNT showed a dose–response inhibition of AChE activity with IC50 values of 4, 17, 156, and 96 mgL^−1^, respectively. The inhibition by nanoparticles was primarily caused by adsorption or interaction with the enzyme [66].

All these findings about the the sensitivity of AChE to several classes of contaminants other than organophosphate and carbamate compounds need to be taken into account for the proper application of this biomarker in environmental and occupational medicine. In fact, in most cases mixed exposures are observed. It is worth noting that not only different compounds may reach levels of significance in terms of anticholinesterase effect, but, moreover, combinations of different chemical classes can exert additive or synergistic inhibitory effect on AChE activity. This suggests the need to reconsider the applicability of AChE in biomonitoring and risk assessment in areas contaminated by several classes of pollutants. In these cases the usefulness of this biomarker could be that of providing an integrative measurement of the overall neurotoxic risk posed by the whole burden of bioavailable contaminants present in the environment.

## 6. Noncatalytic Functions of ACHE and Organophosphate Sensitivity

Research in the last twenty years indicates additional functions of AChE besides its catalytic activity and its role in terminating neurotransmission at cholinergic synapses. Different isoforms of AChE have been shown to affect cell proliferation, differentiation, and responses to various stresses. AChE appears to play an important role in axonal outgrowth [67], synaptogenesis [68], cell adhesion [69], neuronal migration [70], hemopoietic stress responses [71], and apoptosis [72]. These functions are largely independent of the enzymatic ability to hydrolyze acetylcholine [9]. The mechanisms underlying these important noncatalytic functions are to be explored; however, they seem to involve alternatively spliced AChE variants in several tissues. 

It is known that multiple stress stimuli involve increased ratio between AChE-R and AChE-S in brain and blood cells [9, 71].

In brain AChE-S is the main isoform in physiologic conditions, but the normally rare AChE-R variant can occur after exposure to physical stress or anticholinesterase drugs [73]. In general, under normal conditions, the splicing factors SC35 and ASF/SF2 balance each other and regulate the splicing of AChE, raising the level of the AChE-S form and lowering the level of the AChE-R form [9]. During stress, the upregulated SC35 induces an imbalancing of the dynamic ratio of AChE-S/R variants by shifting the splicing of AChE-S form to the AChE-R form through interacting with a specific exonic splicing enhancer [74]. 

The two AChE splice variants, R and S, share distinct functions in development and repair in the brain: the AChE-R isoform, preferentially induced by injury, appears to promote repair and protect against neurodegeneration, while overexpression of the more abundant synaptic isoform, AChE-S, enhances susceptibility to neurotoxicity.

Recently Jameson et al. [75] suggested that the nonenzymatic functions of AChE splice variants are a target for the developmental neurotoxicity of organophosphates. As demonstrated in animal models organophosphate compounds are able to induce developmental neurotoxicity at doses that do not elicit any signs of systemic intoxication and even at exposures below the threshold for inhibition of AChE [76–78]. In humans links between organophosphate exposure during pregnancy and deficits in fetal growth and neurocognitive development in children were observed [79]. These findings have led to the restriction of the household use of some organophosphate insecticides in some countries. However, the mechanisms and consequences of organophosphate-induced developmental neurotoxicity remain a major environmental concern.

Organophosphates are known to increase the overall expression of AChE and to alter the relative expression of AChE-R and AChE-S in mammal adult brain [80, 81]. On the other hand during the developmental period, exposures to organophosphate elicit an AChE pattern associated with progressive neurotoxicity characterized by coinduction of both AChE-R and AChE-S at concentrations of exposure below the threshold for inhibition of AChE catalytic activity [75]. As pointed out by Jameson et al. [75], AChE variants may participate in and be predictive of the relative developmental neurotoxicity of organophosphates, including long-term cognitive impairment [82]. 

Recently, organophosphate exposure was found to be associated with an increased risk of Alzheimer's disease in workers exposed to these compounds [83] and, in addition, with an increased risk of Alzheimer's disease in children [84]. On the basis of the study of Darreh-Shori et al. [85] who have explored the roles of the two AChE variants in the Alzheimer disease, it is possible to hypothesize the involvement of the AChE different spicing isoforms in the organophosphate association with Alzheimer's disease in exposed individuals.

Interestingly, all these results point out the need of analysing AChE gene splice variants that may be important in the mechanisms or outcomes of organophosphate-induced developmental neurotoxicity and not just the total activity of the protein product. Moreover, they open new perspectives for the potential use of AChE gene expression in biomonitoring and risk assessment. In perspective, the study of AChE gene splice variants, of their functions, and of the pollutants-induced alterations in their expression pattern could contribute (1) to detect exposure to pollutant concentrations that do not elicit any signs of systemic intoxication and AChE inhibition in adults but that are able to induce long-term effects on developmental stages; (2) to define new threshold exposure levels that protect the organism against adverse effects at all life stages; (3) to characterize new biomarkers of susceptibility.

## 7. Conclusions

AChE represents one of the first validated biomarkers in environmental and occupational medicine and its use is increased in the last two decades. However, recent findings indicate new potentialities of AChE in human biomonitoring. The sensitivity of AChE activity to other classes of chemicals, including emerging pollutants such as nanomaterials, suggests the usefulness of this biomarker for providing an integrative measurement of the overall neurotoxic risk arising from the whole burden of bioavailable contaminants in areas contaminated by several classes of pollutants. Moreover, the study of the expression of AChE splice variants, their role in the neurotoxicity of organophosphate, contributes to the development of AChE gene expression as a new biomarker of susceptibility to improve the understanding of environmental and occupational health.
